# The efficacy of saffron supplementation on inflammation and oxidative stress in patients with type-2 diabetes mellitus: a meta-analysis

**DOI:** 10.3389/fendo.2025.1634982

**Published:** 2025-08-06

**Authors:** Lulu Qin, Nini Hu, Kejing Wang, Lin Chen

**Affiliations:** ^1^ Department of Pharmacy, Women and Children’s Hospital of Chongqing Medical University, Chongqing, China; ^2^ Department of Pharmacy, Chongqing Health Center for Women and Children, Chongqing, China

**Keywords:** saffron, type-2 diabetes mellitus, inflammation, oxidative stress, meta-analysis

## Abstract

**Background:**

In this study, a meta-analysis was performed to evaluate the changes in the levels of inflammation and oxidative stress levels in type 2 diabetes mellitus (T2DM) patients after saffron supplementation.

**Methods:**

A comprehensive and systematic investigation was carried out to identify relevant studies on PubMed, Web of Science, The Cochrane Library, and EMBASE databases. The investigation aimed to find studies that tested the efficacy of saffron supplements in reducing inflammation and oxidative stress in people with type 2 diabetes. The data were synthesized using Revman 5.4 software to perform a meta-analysis.

**Results:**

A total of five relevant articles involving 261 patients were included. The meta-analysis results demonstrated a significant reduction in TNF-α levels following saffron supplementation compared to the placebo group. However, no significant changes were observed in the levels of CRP levels (SMD: 0.01, 95% CI: −0.52 to 0.05, p = 0.97), IL-10 levels (SMD: 0.31, 95% CI: −0.05 to 0.67, p = 0.09), IL-6 levels (SMD: −0.28, 95% CI: −0.79 to 0.24, p = 0.29), MDA levels (SMD: 0.01, 95% CI: −0.28 to 0.30, p = 0.95), and TAC levels (SMD: −0.09, 95% CI: −0.42 to 0.24, p = 0.59).

**Conclusion:**

The findings of this meta-analysis provide compelling evidence to support the efficacy of saffron supplementation in reducing the effects of TNF-α levels in individuals with T2DM. Nevertheless, the present body of research is still limited in its ability to establish the precise impact of saffron supplementation on oxidative stress and other inflammatory factors in T2DM patients.

## 1 Introduction

Type 2 diabetes mellitus (T2DM) is a prevalent endocrine disorder characterized by hyperglycemia resulting from insulin resistance or inadequate insulin secretion (1). The increasing prevalence of T2DM has made it a global public health concern (2). Notably, T2DM imposes a substantial financial burden on patients and adversely affects their quality of life. Moreover, the condition is associated with a range of complications, including cardiovascular disease, chronic kidney disease, retinopathy, and peripheral neuropathy (3, 4). Currently, T2DM treatment involves pharmacological interventions and lifestyle modifications. However, the efficacy of pharmacotherapy is limited by poor patient adherence and side effects such as obesity, gastrointestinal reactions, and hypoglycemia (5). Exploring the complementary treatment of T2DM has become a new research hotspot.

Accumulating evidence indicates that elevated blood glucose levels in patients with T2DM can trigger inflammation and oxidative stress (6, 7). Inflammation and oxidative stress have emerged as significant contributors to the onset and progression of T2DM and its associated complications (8). In T2DM patients, there is often a concurrent increase in the levels of various inflammatory factors such as interleukin (IL), tumor necrosis factor α (TNF-α), and C-reactive protein (CRP) (9). Elevated blood glucose levels can trigger the production of reactive oxygen species (ROS) in individuals with T2DM leading to oxidative stress in islet beta cells and a consequent decline in insulin secretion (10). Moreover, a complex interplay exists between inflammation and oxidative stress in T2DM patients. Inflammatory processes can increase ROS levels, thereby exacerbating oxidative stress and causing alterations in malondialdehyde (MDA) and total antioxidant capacity (TAC) levels (11). Conversely, oxidative stress can damage adipose tissue, prompting the release of adipocytokines, such as TNF-α and IL-6, which induce inflammation and exacerbate insulin resistance (12). Hence, mitigating inflammation and improving oxidative stress levels in T2DM patients is crucial for successfully treating and preventing its related complications.

Saffron (*Crocus sativus* L.), also known as crocus, is an herb with a history spanning thousands of years. It contains active substances such as safranal, picrocrocin, and crocetin (13). Saffron has a variety of pharmacological effects at the same time, including anti-oxidative stress, anti-inflammatory, lipid lowering, and anticancer effects (14, 15). In recent years, several studies have reported that saffron supplementation can potentially reduce hyperglycemia and improve insulin sensitivity (16, 17). However, the impact of saffron supplementation on inflammation and oxidative stress in T2DM patients remains unclear. Consequently, this meta-analysis aims to provide evidence-based findings on the effect of saffron supplementation in alleviating inflammation and oxidative stress, particularly in T2DM patients.

## 2 Methods

The present systematic review and meta-analysis adhered to the Preferred Reporting Items for Systematic Reviews and Meta-Analysis (PRISMA) statement guideline (18).

### 2.1 Search strategy

A comprehensive search for randomized controlled trials exploring the effect of saffron supplementation on inflammation and oxidative stress in T2DM patients was conducted systematically across multiple academic databases, including PubMed, Web of Science, The Cochrane Library, and EMBASE. The search was initiated from the inception of each database in April 2025. A combination of MeSH terms and free-text words was used for the search. The search terms were as follows: ((((Crocus[MeSH Terms]) OR (Saffron crocus[Title/Abstract]) OR (Crocus sativus[Title/Abstract]) OR (Saffron[Title/Abstract])) AND (((Diabetes Mellitus, Type 2[MeSH Terms]) OR (Stable diabetes mellitus[Title/Abstract]) OR (Diabetes mellitus*[Title/Abstract]))). Additionally, the references within the retrieved literature were screened to identify more comprehensive relevant studies.

### 2.2 Inclusion and exclusion criteria

Inclusion criteria:

(P) Participants: Patients must meet the diagnostic criteria for type 2 diabetes outlined by the American Diabetes Association (19) and be aged ≥ge years.
(I) Interventions: Saffron and its extracts.
(C) Controls: The placebo was similar to the intervention group regarding size, taste, color, shape, smell, and dispensing container. Its components will consist of either starch or maltodextrin.
(O) Outcomes: The outcomes included oxidative stress and inflammatory biomarkers.
(S) Study design: Randomized controlled trials.

Exclusion criteria:

1. Duplicate publications.
2. Animal experiments or review articles.
3. Incomplete data or inaccessible data.

### 2.3 Data extraction

Two researchers screened the literature separately according to inclusion and exclusion criteria. A third researcher is required to participate in the discussion to decide whether to include the literature in cases of disagreement. Two researchers also independently extracted data, including the first author and publication year, country, sample size, age, specific interventions in the trial and control groups, drug dosage, and intervention duration. The extracted data were cross-checked; any discrepancies were resolved by a third researcher.

### 2.4 Quality assessment

Using the Cochrane Risk of Bias Assessment Tool (20), two researchers comprehensively evaluated the methodological quality of the included studies based on seven domains: random sequence generation, allocation concealment, blinding (of both the researchers and the participants and for outcome measurers), completeness of outcome data, selective reporting, and other sources of bias. The risk of bias was categorized into three levels: “low risk of bias,” “unclear risk,” and “high risk of bias.” To ensure the reliability of the results, cross-validation was performed, and a third researcher was consulted to resolve any disagreements.

### 2.5 Data synthesis and statistical analysis

Meta-analyses were conducted using Review Manager software (version 5.4). For continuous variables, the standard mean difference (SMD) and its corresponding 95% confidence interval (CI) were employed as pooled statistics. The heterogeneity of the collected data was quantitatively assessed using the χ2 test and *I^2^* statistic. A random-effects model was used for pooled analyses if substantial heterogeneity was indicated (*p < 0.1 or I² ≥ 50%*) Conversely, if there was no significant heterogeneity, a fixed-effects model was utilized in the combined analysis.

## 3 Results

### 3.1 Literature search

**Figure 1:**
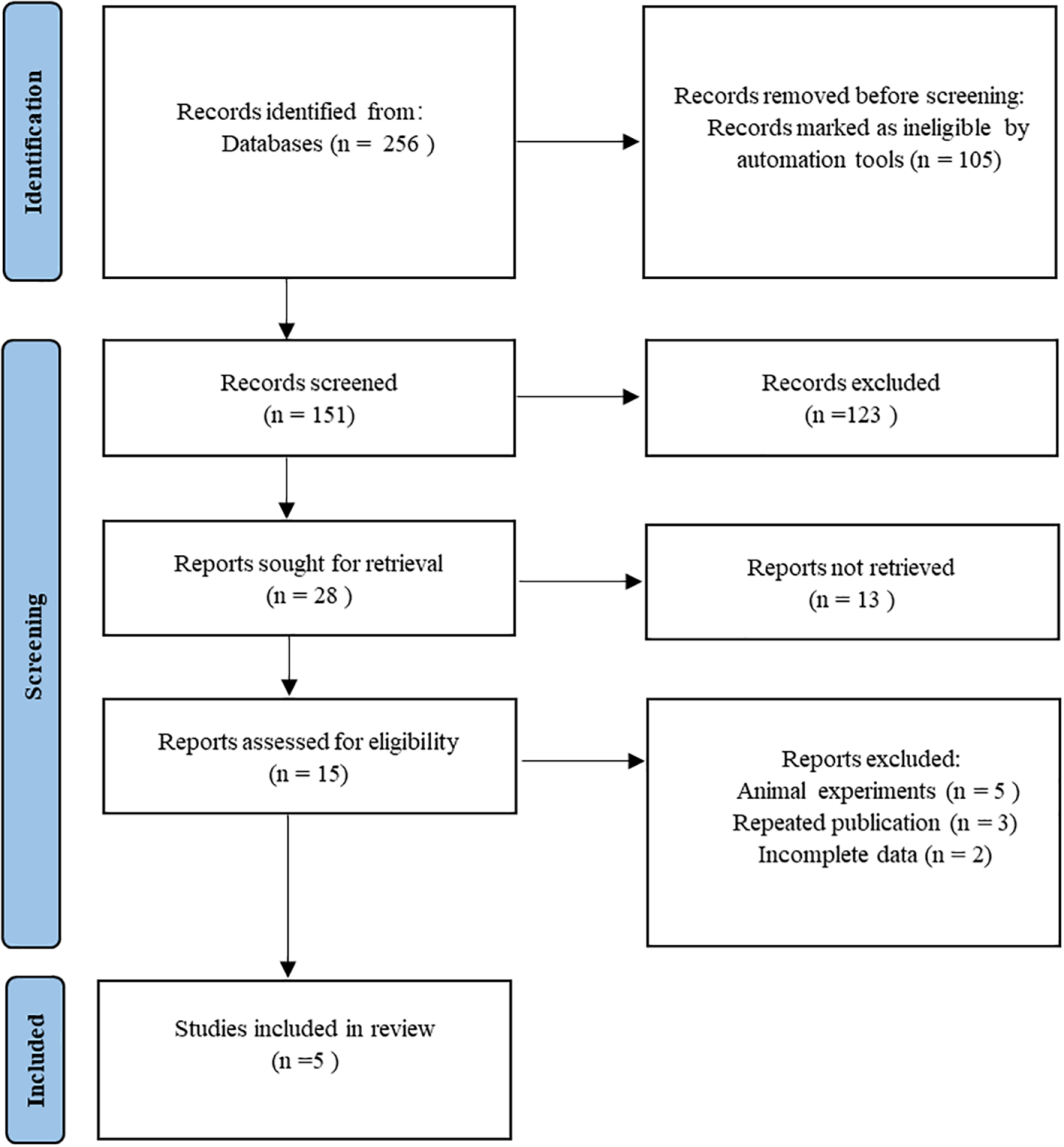
Flowchart for inclusion of studies.

A total of 256 related studies were initially retrieved from the databases. EndNote software was used to remove 105 duplicate records. Subsequently, 123 articles were screened by reading titles and titles. After the initial screening, full texts were carefully read, including 13 reviews, 5 animal studies, 3 repeated publications, and 2 incomplete data. Finally, five studies (21–25) were included. The flowchart of literature screening is shown in **Figure 1**.

### 3.2 Characteristics of included studies

The included studies were all from Iran, and the literature was published from 2019 to 2022. The total sample size across all studies was 261 participants, with the smallest individual study sample size being 16 and the largest 80. All five studies were randomized controlled trials of double-blind placebo. The dosage of saffron supplements ranged from 6 to 400 mg. The duration of intervention was 8–12 weeks. The outcome indicators were oxidative stress markers (MDA and TAC) and inflammatory factors (CRP, IL-6, IL-10, and TNF-ɑ). The essential characteristics of the literature are shown in **Table 1**.

**Table 1:** Main characteristics of included studies.

Study, year	Country	Registration number	Participants		Ages		Intervention		Dose	Duration (week)
			Treatment group	Control group	Treatment group	Control group	Treatment group	Control group		
Behrouz et al., 2021 (25)	Iran	NCT04163757	22	22	57.08 ± 7.41	59.86 ± 9.46	Crocin	Placebo	6 mg	12
Ebrahimi et al., 2019 (24)	Iran	IRCT201510259472N9	40	40	55.2 ± 7.3	53 ± 10.6	Saffron	Placebo	100 mg	12
Mobasseri et al., 2020 (23)	Iran	IRCT20090609002017N24	30	27	50.57 ± 9.88	51.63 ± 11.30	Saffron	Placebo	100 mg	8
Shahbazian et al., 2019 (21)	Iran	IRCT2015110219739N1	32	32	53.5 ± 9.9	52.4 ± 13	Saffron	Placebo	30 mg	12
Rajabi et al., 2022 (22)	Iran	–	8	8	54.12 ± 7.37	56.87 ± 5.11	Saffron	Placebo	400 mg	8

### 3.3 Quality assessment

**Figure 2:**
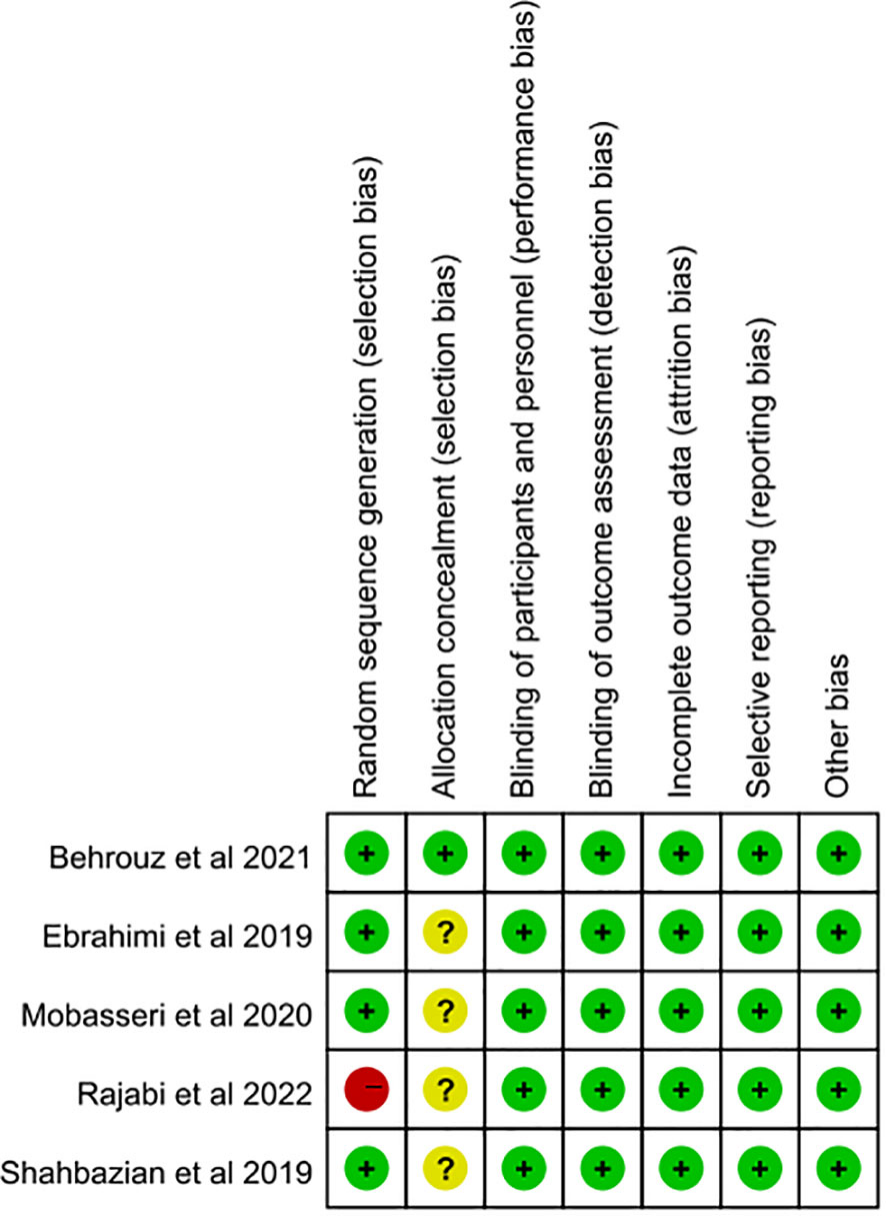
Risk of bias assessment of included studies according to the Cochrane collaboration’s tool.

The random sequence generation was judged to be at low risk of bias in four studies (21, 23–25). One piece of literature (22) was assessed as having a high risk of bias due to its randomization method. Regarding the allocation concealment, one piece of literature (25) was classified as low-risk bias, while the remaining four pieces (21–24) provided insufficient details and were thus classified as unclear. Notably, all five studies (21–25) were double-blind and contained complete outcome index data showing no evidence of selective outcome reporting. As a result, they were all deemed to have a low risk of bias. Overall, the methodological design of the included RCTs was quite rigorous. For further details regarding the risk assessment of bias, refer to **Figure 2**.

### 3.4 The effect of saffron supplementation on serum levels of CRP

**Figure 3:**
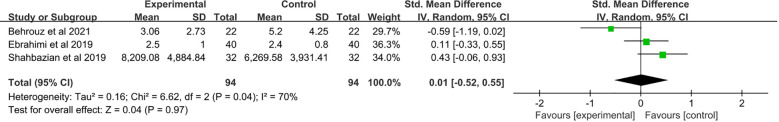
Forest plot: The efficacy of saffron supplementation on serum levels of C-reactive protein.

A total of three studies (21, 24, 25) were included. Meta-analysis showed no significant change in CRP levels after saffron supplementation compared with placebo (SMD: 0.01, 95% CI: −0.52 to 0.05, p = 0.97) (**Figure 3**).

### 3.5 The effect of saffron supplementation on serum levels of TNF-ɑ

**Figure 4:**
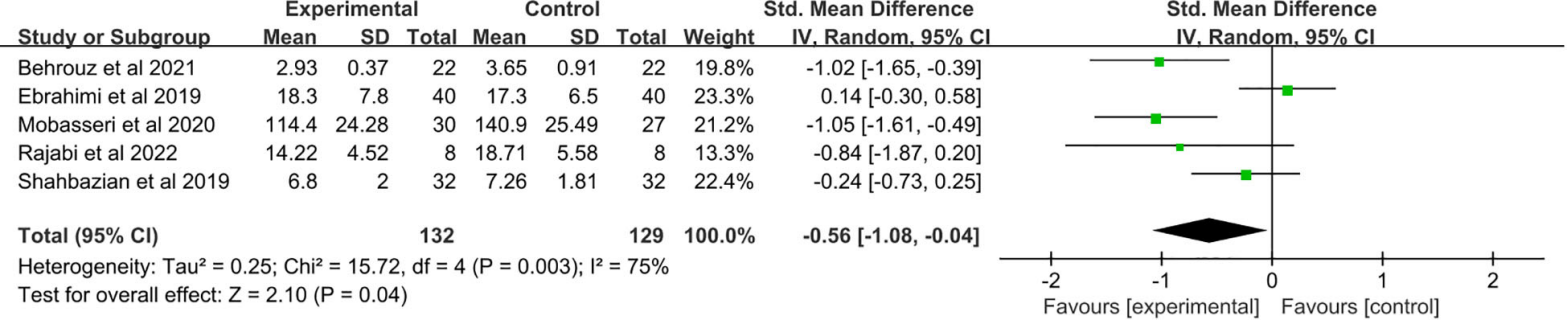
Forest plot: The efficacy of saffron supplementation on serum levels of tumor necrosis factor α.

A total of five studies (21–25) were included. Saffron supplementation was associated with a significant reduction in TNF-α levels compared with the placebo group (SMD: −0.56, 95% CI: −1.08 to −0.04, p = 0.04) (**Figure 4**).

### 3.6 The effect of saffron supplementation on serum levels of IL-10

**Figure 5:**
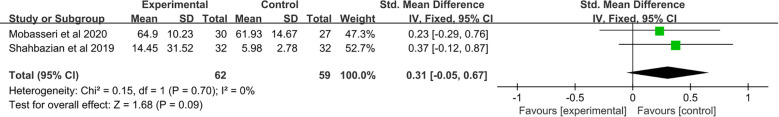
Forest plot: The efficacy of saffron supplementation on serum levels of interleukin-10.

A total of two studies (21, 23) were included. No significant change in IL-10 levels was observed after saffron supplementation compared with the placebo group (SMD: 0.31, 95% CI: −0.05 to 0.67, p = 0.09) (**Figure 5**).

### 3.7 The effect of saffron supplementation on serum levels of IL-6

**Figure 6:**
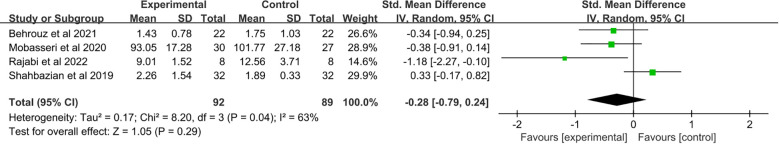
Forest plot: The efficacy of saffron supplementation on serum levels of interleukin-6.

A total of four studies (21–23, 25) were included. Meta-analysis showed no significant change in IL-6 levels after saffron supplementation compared with placebo (SMD: −0.28, 95% CI: −0.79 to 0.24, p = 0.29) (**Figure 6**).

### 3.8 The effect of saffron supplementation on serum levels of MDA

**Figure 7:**
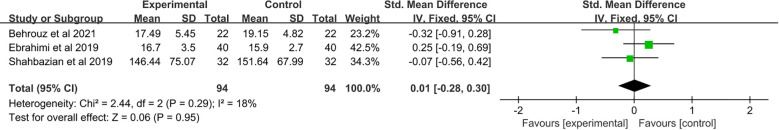
Forest plot: The efficacy of saffron supplementation on serum levels of malondialdehyde.

A total of three studies (21, 24, 25) were included, which revealed no significant changes in MDA levels (SMD: 0.01, 95% CI: −0.28 to 0.30, p = 0.95) (**Figure 7**).

### 3.9 The effect of saffron supplementation on serum levels of TAC

**Figure 8:**
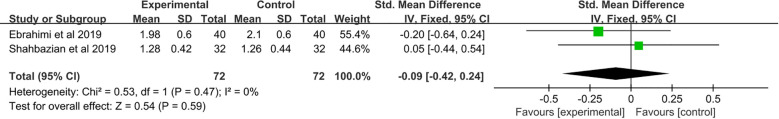
Forest plot: The efficacy of saffron supplementation on serum levels of total antioxidant status.

A total of two studies (21, 24) were included. Meta-analysis showed no significant change in TAC levels after saffron supplementation compared with placebo (SMD: −0.09, 95% CI: −0.42 to 0.24, p = 0.59) (**Figure 8**).

### 3.10 Subgroup analysis and publication bias analysis

There are too few studies included to conduct subgroup analysis and publication bias testing.

### 3.11 Sensitivity analysis

**Figure 9:**
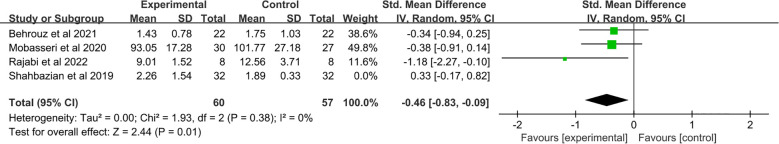
Forest plot: The efficacy of saffron supplementation on serum levels of interleukin-6 (sensitivity analysis).

In conducting a sensitivity analysis, we employed the one-by-one exclusion method. The results showed that in studies related to IL-6, when one study (21) was excluded, compared to the control group, supplementation with saffron resulted in a decrease in IL-6 levels (SMD: −0.46, 95% CI: −0.83 to −0.09, p = 0.01) (**Figure 9**). When p < 0.05, the difference is statistically significant, which may be related to the negative results of the excluded studies. However, the research results of other indicators did not show significant changes in the combined effect quantity, and the results were relatively robust.

## 4 Discussion

Elevated blood glucose levels drive the progression of T2DM and induce a series of complications, including neuropathy, retinopathy, atherosclerosis, and kidney disease, which pose a significant threat to patients’ quality of life and survival (26, 27). Hence, controlling blood glucose levels is the key to treating T2DM and preventing these complications (28, 29). Therefore, blood glucose levels can be regulated by improving inflammation and oxidative stress in T2DM patients to achieve therapeutic goals. It is worth noting, however, that the current glucose-lowering drugs have certain limitations, and their efficacy in preventing complications is suboptimal (30).

This meta-analysis investigated the effects of saffron supplementation on inflammation and oxidative stress in patients with T2DM. The results of the meta-analysis indicated that saffron supplementation exerted a beneficial effect in reducing the influence of TNF-ɑ in T2DM patients but did not significantly impact other markers of inflammation and oxidative stress. A meta-analysis of five studies (21–25) further supported the efficacy of saffron supplements in lowering serum TNF-ɑ levels. In addition, after excluding one negative study (21), the meta-analysis results of the remaining three studies (22, 23, 25) demonstrated that saffron supplements also contributed to reducing serum IL-6 levels. Notably, relatively few studies have examined the associations between inflammatory factors (IL-10 and CPR) and markers of oxidative stress (MDA and TAC) with T2DM. As a result, the pooled results have not yet reached statistical significance. However, accumulating evidence suggests that inflammation and oxidative stress are indeed closely linked to the development of T2DM (31–33). Oxidative stress occurs when ROS accumulate excessively and exceeds the body’s antioxidant capacity (34). This, in turn, can lead to the destruction of beta cells and impair insulin secretion through various molecular mechanisms (35, 36), while also inducing pancreatic islet cell apoptosis, leading to beta cells loss or death (37, 38).

Furthermore, notably, oxidative stress can exert varying degrees of damage to insulin signal transduction, disrupt insulin signaling pathways, and ultimately give rise to insulin resistance (39). As the primary mediators, the inflammatory factors TNF-α and IL-6 cooperated with other factors to inhibit or damage insulin secretion by pancreatic islet beta cells and simultaneously reduce the activity of insulin receptors, thus leading to insulin resistance (40). During the inflammatory response, various inflammatory factors promote each other’s expression. CRP, an acute-phase protein produced by the liver, promotes the inflammatory response by increasing the synthesis of TNF-α and IL-6, which in turn contributes to insulin resistance (41). Conversely, TNF-α also induces CRP expression by upregulating IL-6 (42). In addition, studies have shown that in a hyperglycemic state, TNF-α concentrations are significantly higher compared with other inflammatory factors, which also exhibit significant elevations (43). This suggests that the inflammatory factor TNF-α is an important indicator that T2DM patients are in a hyperglycemic state. Saffron has both antihyperglycemic and insulin-sensitizing effects (16, 17). Notably, changes in serum concentrations of TNF-α can serve as a marker for saffron’ s hypoglycemic and anti-inflammatory efficacy. As a natural source of antioxidants, saffron has the capacity to reduce inflammatory factors and alleviate oxidative stress (44, 45). Its antioxidant properties ameliorate oxidative stress by lowering intracellular ROS levels, thereby reducing ROS-mediated cellular damage. Furthermore, its bioactive components can decrease the production of IL-6, TNF-α, and other factors, inhibit the activation of additional inflammatory factors under hyperglycemic conditions, and thus suppress inflammation (46). Following the amelioration of inflammation and oxidative stress in T2DM patients, insulin sensitivity and β-cell function can be enhanced, the insulin signaling pathway is preserved, and hyperglycemia can be better controlled (47).

Current evidence indicates that saffron can significantly reduce TNF-α levels in patients with type 2 diabetes mellitus (T2DM), a mechanism potentially mediated by the inhibition of the NF-κB pathway (48, 49). As a pro-inflammatory cytokine, TNF-α reduction is likely to alleviate insulin resistance and cardiovascular risks (48). Some RCTs have shown that crocin (at a dosage of ≥fs mg/day) can simultaneously decrease CRP and IL-6 (25, 50), whereas other studies failed to observe significant improvements in markers such as IL-6 and CRP (48). Such discrepancies may be associated with differences in dosage, intervention duration, and active ingredients (25, 50). Daily supplementation with ≥it mg of crocetin (the main active component of saffron) can significantly lower inflammatory markers (hs-CRP, TNF-α, NF-κB) and oxidative stress indices (25). Short-term interventions show no significant effects on inflammatory indicators, while those lasting ≥as weeks can stably reduce CRP, TNF-α, and fasting blood glucose (25). In an 8-week randomized controlled trial, saffron significantly reduced hyperglycemia and hyperlipidemia, as well as improved liver function in patients with T2DM. Additionally, saffron significantly ameliorated depression, sleep quality, and overall quality of life in T2DM patients (17).

Currently, there is a lack of direct comparative studies comparing saffron with standard anti-inflammatory drugs. Indirect evidence suggests that saffron can reduce pain scores and the number of swollen joints in patients with arthritis, but it does not significantly modulate serum markers, such as TNF-α and hs-CRP (51), implying that its anti-inflammatory mechanism may differ from that of conventional drugs.

Caution is warranted in clinical integration: although the reduction in TNF-α may be beneficial for cardiac protection (48), the effects of saffron on other inflammatory factors (e.g., IL-6) and oxidative stress indices (e.g., MDA, TAC) are inconsistent (51), and its efficacy needs to be evaluated on an individual patient basis. A decrease in TNF-α levels may be associated with improvements in insulin sensitivity and cardiovascular risk; however, existing studies have not elucidated its relevance to clinical endpoints (e.g., reduced cardiovascular events) (48). Although saffron reduces TNF-α and fasting blood glucose, it exerts significant impact on other cardiovascular risk factors such as blood lipids and blood pressure (48). Furthermore, one RCT found that saffron supplementation did not alter TNF-α levels but significantly reduced waist circumference and MDA (24). These findings further indicate that the clinical efficacy of saffron requires verification through large-sample size, long-term studies.

## 5 Limitations

The present study has generated several insightful findings; however, its inherent limitations must be acknowledged. First, all the studies included in this investigation were conducted in a single country, which limits the generalizability of the findings to other populations. Second, inflammation and oxidative stress in T2DM patients are affected by multiple factors, which may introduce bias into the results. Third, the limited number of studies on each outcome measure precluded the possibility of subgroup analysis and publication bias assessments. Consequently, future research should include larger sample sizes, encompass diverse ethnicities, and adopt a multicenter design. Fourth, the indicators examined in this study represent only a small fraction of the numerous markers of inflammation and oxidative stress emphasizing the need to include more comprehensive markers in future analyses.

## 6 Conclusion

In conclusion, this meta-analysis provides evidence-based support for using saffron supplements. Saffron supplements in T2DM patients may avoid many adverse reactions and is less likely to induce drug resistance. At the same time, it has other health benefits such as lowering blood pressure and improving qi and blood circulation. This meta-analysis showed that saffron supplementation had a beneficial effect on the inflammatory factor TNF-α in T2DM patients. However, evidence remains insufficient to confirm the specific effects of saffron supplementation on oxidative stress and other inflammatory factors in T2DM patients.

## Data Availability

The original contributions presented in the study are included in the article/supplementary material, further inquiries can be directed to the corresponding author/s.
